# Post-traumatic Pseudocyst of the Spleen: A Report of a Rare Case

**DOI:** 10.7759/cureus.82386

**Published:** 2025-04-16

**Authors:** Aravind Kumar, Alexander Mecheri Antony

**Affiliations:** 1 General Surgery, Sree Balaji Medical College & Hospital, Chennai, IND; 2 Surgery, Sree Balaji Medical College & Hospital, Chennai, IND

**Keywords:** road traffic injuries, splenic pseudocyst, splenic trauma, subcapsular splenic hematoma, total splenectomy, traumatic splenic rupture

## Abstract

Splenic pseudocysts are multifactorial in etiology, with trauma being the most common causative factor, wherein an intrasplenic hematoma forms and subsequently liquefies. They can also develop as a result of infections - both local and systemic - through an inflammatory process and tissue necrosis. Pancreatitis-induced splenic pseudocysts, although exceedingly rare, arise either from the direct extension of pancreatic inflammation, enzymatic autodigestion of the pancreas, or the close anatomical relationship between the pancreas and spleen. Splenic pseudocysts are diagnosed by demonstrating their size, location, and internal septations or calcifications on imaging modalities such as computed tomography (CT) and magnetic resonance imaging. Although less sensitive than CT at discerning subtle details, ultrasonography plays a major role in initial and longitudinal monitoring, particularly due to its lack of radiation exposure, which is beneficial for certain patient populations, including pregnant or pediatric patients.

## Introduction

Splenic pseudocysts, also referred to as secondary splenic cysts, are acquired cystic lesions that lack a true epithelial lining [1]. They account for approximately 80% of all splenic cystic lesions. The most common cause is splenic trauma (around 75%), often due to overlooked injuries or, more rarely, pancreatitis [2], followed by splenic infarction and local inflammatory processes, such as intrasplenic pancreatic pseudocysts [3]. These cysts represent the majority (about 80%) of benign splenic cysts. Clinically, most splenic pseudocysts are asymptomatic and are often discovered incidentally during imaging for unrelated reasons [4]. The majority arise secondary to abdominal trauma, but they can also result from infections like tuberculosis, malaria, or infectious mononucleosis. Cysts smaller than 5 cm typically remain asymptomatic, while larger cysts (>8 cm) are more likely to cause symptoms due to progressive enlargement and compression of nearby structures [5]. Symptoms may include pain in the left upper quadrant or epigastric region, early satiety due to stomach compression, fever from abscess formation, or hypersplenic sequestration.

Splenic pseudocysts are considered the end-stage result of splenic injury, often following trauma or infarction. They develop due to liquefactive necrosis and cystic degeneration. Histologically, these cysts are characterized by the absence of an epithelial lining [5]. An ultrasound typically shows well-circumscribed cystic lesions with heterogeneous internal echoes, often due to hemorrhagic debris [5]. A computed tomography (CT) scan demonstrates a hypoattenuating, well-defined lesion within the spleen, with sharp borders and a thin fibrous wall. Calcifications may be present. No internal or peripheral enhancement is noted. Magnetic resonance imaging (MRI) reveals sharply demarcated cystic lesions with variable internal signal characteristics: T1 shows variable signal intensity depending on content, whereas T2 typically shows very high signal intensity. Small, asymptomatic cysts generally require no intervention or follow-up. Larger or symptomatic cysts are usually managed surgically, either by total splenectomy or spleen-preserving techniques such as partial splenectomy, marsupialization, or fenestration [5-8]. In some cases, percutaneous drainage may be considered. Complications are rare but may include hemorrhage, rupture, or infection. Other conditions that may mimic splenic pseudocysts on imaging include primary (congenital) splenic cysts, infections, splenic hydatid cysts, bacterial splenic abscesses, other congenital cystic anomalies, splenic lymphangioma, splenic hemangioma, and cystic metastases to the spleen. 

## Case presentation

A 19-year-old male patient came with chief complaints of abdominal pain for one year, with a dull, aching type of pain and on-and-off symptoms, including nausea and vomiting - two to three episodes over the past three days, non-bilious and not blood-stained. The patient also presented with complaints of bloating for the past six months, on and off, and a history of low-grade fever for one week, intermittent in nature. There was a history of trauma - road traffic accident with blunt injury to the abdomen - 10 years ago, which was conservatively managed.

The patient has no comorbidities or previous surgical history. On clinical examination, a mass approximately 8 cm × 7 cm in size was palpable over the left hypochondrium and epigastric regions. The plane of swelling was found to be intra-abdominal, confirmed by head-raise and leg-raise tests.

**Figure 1:**
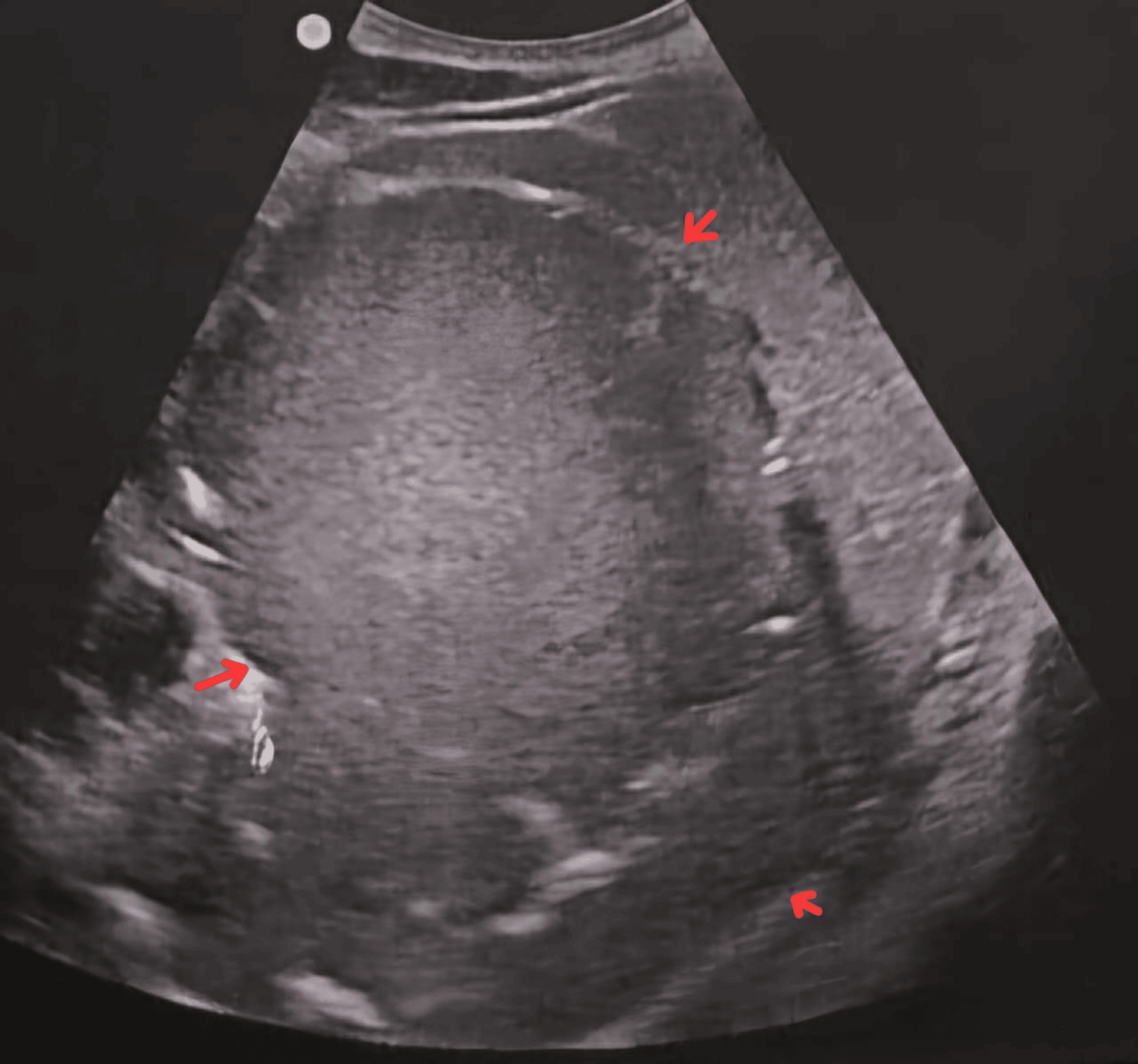
Ultrasonography of the abdomen This image shows a large cystic lesion over the splenic region (red arrows) with laceration over the midpole of the spleen.

**Figure 2:**
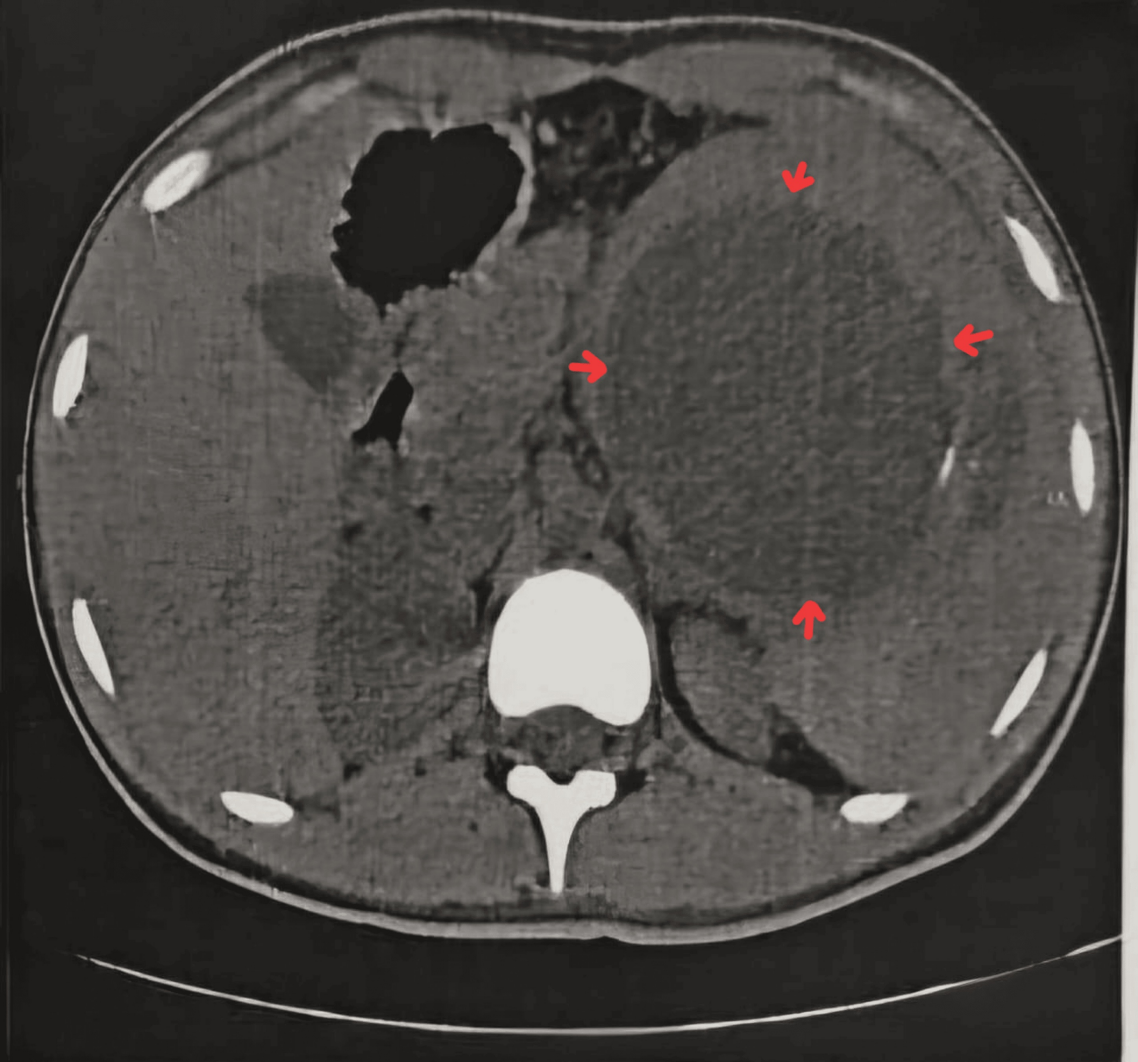
CECT of the abdomen (axial view) This image shows gross splenomegaly with a large cystic lesion (red arrows) measuring approximately 1500 cc, with dense internal contents and a few lobulations within the splenic parenchyma at the upper and mid-pole, extending up to the subcapsular location. There is no solid component or enhancement. The cyst is seen abutting the inferior surface of the left lobe of the liver and the tail of the pancreas, with the stomach displaced to the right side, and no encasement of the splenic vessels. CECT, Contrast-Enhanced Computed Tomography

**Figure 3:**
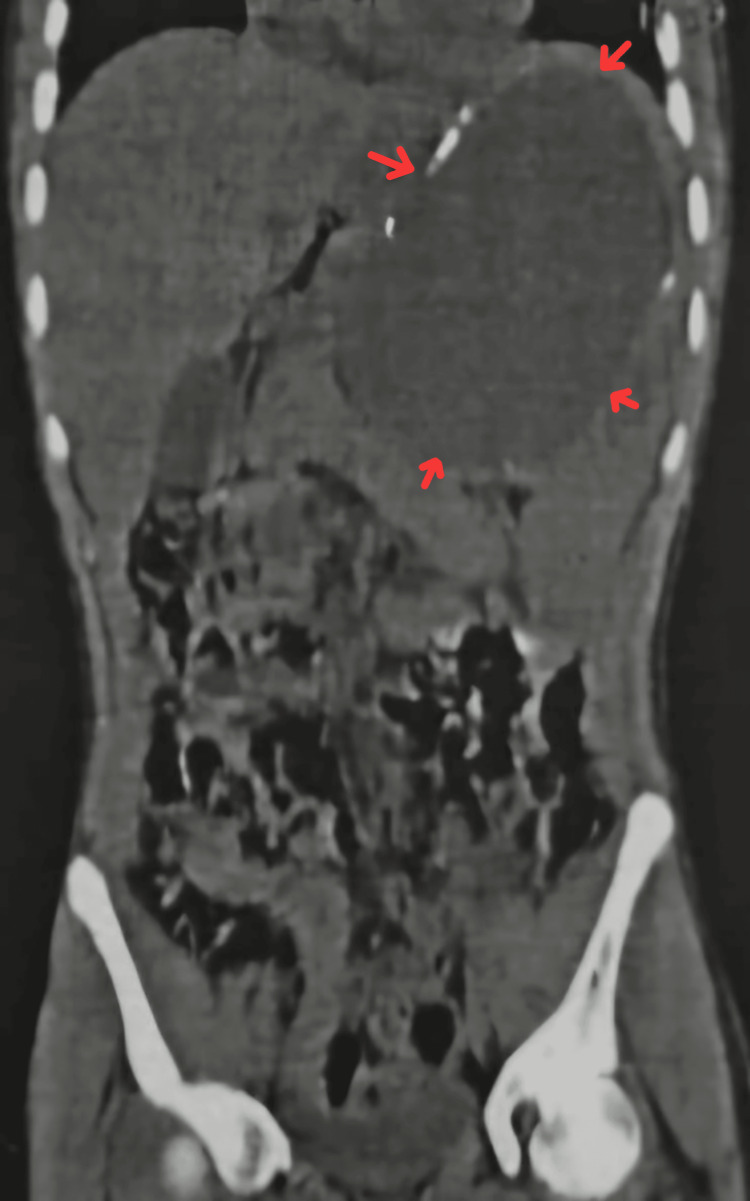
CECT of the abdomen (coronal view) This image shows gross splenomegaly with a large cystic lesion (red arrows), measuring approximately 1500 cc, with dense internal contents and a few lobulations within the splenic parenchyma at the upper and mid-pole, extending up to the subcapsular location. There is no solid component or enhancement. The cyst is seen abutting the inferior surface of the left lobe of the liver and the tail of the pancreas, with the stomach displaced to the right side and no encasement of the splenic vessels. CECT, Contrast-Enhanced Computed Tomography

**Figure 4:**
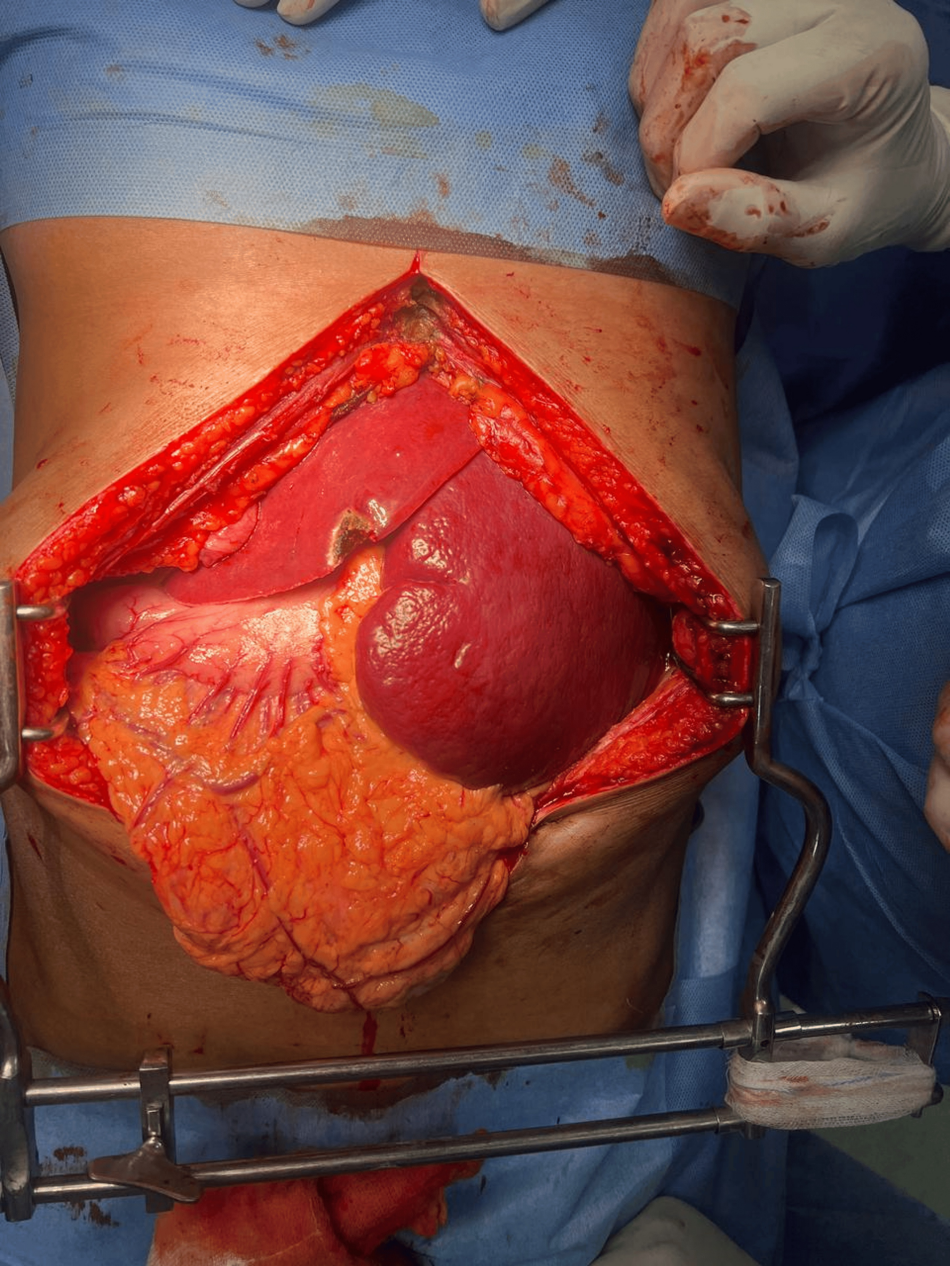
Intra-operative findings This image shows gross splenomegaly with a pseudocyst abutting the inferior surface of the left lobe of the liver and displacing the stomach to the right side, without rupture of the cyst.

**Figure 5:**
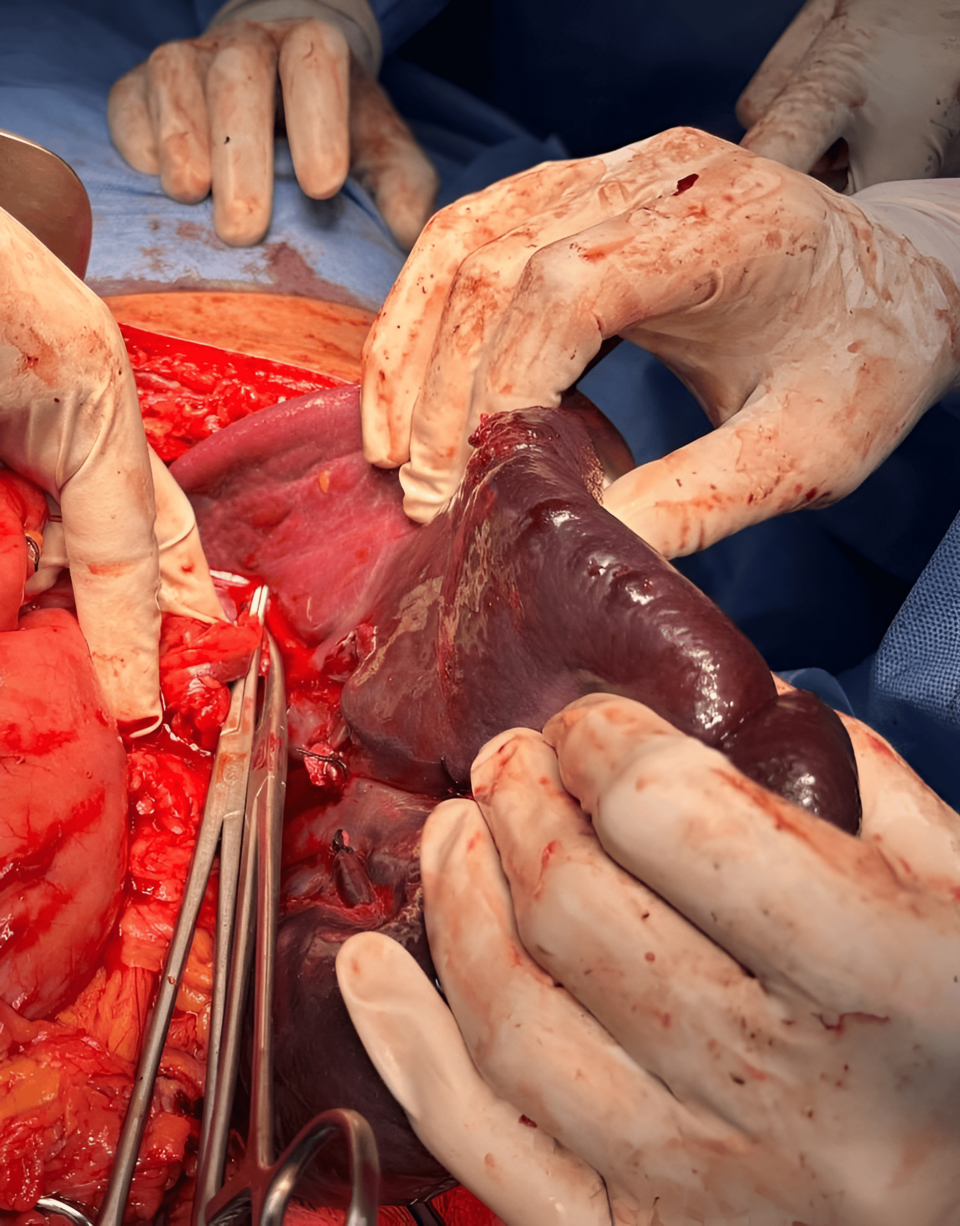
Intra-operative findings This image shows gross splenomegaly with a pseudocyst and splenic injury, with no encasement of the splenic vessels. After clamping the splenic vessels at the hilar region, total splenectomy was performed.

All baseline laboratory investigations were done. The patient tested positive for typhoid. A routine USG abdomen (Figure 1) showed a large cystic lesion over the splenic region, with a laceration over the mid-pole of the spleen. Subsequently, a contrast-enhanced computed tomography (CECT) abdomen (Figures 2-3) was done, which showed gross splenomegaly with a large cystic lesion measuring approximately 1500 cc, containing dense internal contents with a few lobulations within the splenic parenchyma at the upper and mid-pole, extending up to the subcapsular location. There was no solid component or enhancement. The cyst was seen abutting the inferior surface of the left lobe of the liver and the tail of the pancreas, with the stomach displaced to the right side and no encasement of splenic vessels. The patient was planned for surgical excision of the splenic cyst with total splenectomy (Figures 4-5).

## Discussion

The decision to choose the optimal management approach should involve a multidisciplinary team consisting of radiologists, gastroenterologists, and surgeons to provide exhaustive evaluation and individualization of treatment planning, with consideration of the patient’s preferences and comorbidities. The clinical course of traumatic splenic pseudocysts is amenable to non-operative management, based on the tolerance for the pseudocyst to spontaneously involute through gradual reabsorption of the cystic fluid, with restoration of splenic architecture. Image-guided fine needle aspiration can be considered to further characterize the nature of the pseudocyst, as the contents are analyzed and infectious or malignant etiologies are excluded from other cystic lesions. Such information can be gained by examining the aspirated fluid cytologically and by biochemical assessment of amylase and lipase levels in the fluid, in cases in which pancreatic involvement is suspected. Depending upon its size, symptoms, and the presence of complications, splenic pseudocysts are managed according to the patient’s condition. Judicious management of asymptomatic cysts, especially those incidentally found on imaging for unrelated conditions, is recommended with an approach of observation and serial imaging studies for any change in size or morphology [1]. Symptomatic cysts, or those with rapid growth, infection, or bleeding, may require more aggressive intervention, such as percutaneous drainage, laparoscopic fenestration, or, in some cases, splenectomy.

Consequently, however, despite good results and acceptable pregnancy outcomes, careful monitoring for complications, such as rupture, infection, and compression of surrounding structures, is indicated. For splenic pseudocysts that develop secondary to pancreatitis, treatment based on managing the underlying pancreatic inflammation, including pancreatic enzymes, nutritional support, and, in some cases, surgical or endoscopic interventions to relieve pancreatic ductal obstruction of fluid collections, is necessary. Prompt diagnosis of a splenic pseudocyst should therefore be particularly specific, as complications should be prevented [2]. Treatment of pseudocysts of the spleen varies, depending upon the presentation. Given the diverse clinical spectrum of splenic pseudocysts, ranging from incidental asymptomatic findings to potentially morbid symptomatic presentations, management strategies must be carefully tailored to the individual patient, integrating a comprehensive understanding of the cyst's etiology, size, and associated complications to optimize patient-centered care, enhance therapeutic efficacy, and mitigate potential iatrogenic risks.

Splenic pseudocysts tend to rupture and can lead to an almost 90% survival rate with surgical intervention [3]. The development of minimally invasive procedures, such as laparoscopic fenestration and percutaneous drainage, has completely changed the treatment of splenic pseudocysts and given patients less invasive options than open splenectomy, with lower morbidity, shorter hospital stays, and better cosmetic results. Laparoscopic fenestration is a safe and efficient method of treating symptomatic or enlarging splenic pseudocysts by making a wide window in the cyst wall that drains the cyst continuously into the peritoneal cavity. Using this procedure, the chances of recurrence are also reduced, and the symptoms are treated long-term. Although generally unsatisfactory for relieving most cases of temporary cyst symptoms due to rapid recollection of internal pressure, percutaneous drainage guided by CT or ultrasound is a less invasive alternative for patients who are not suitable for surgery, mostly because of comorbidities or contraindications. Despite this, it is important to note that there is a higher risk of recurrence with percutaneous drainage than with laparoscopic fenestration. Therefore, patient selection and long-term follow-up need to be carefully considered. The complete surgical removal of the spleen, splenectomy, is a viable option for the treatment of recurrent or complicated splenic pseudocysts, particularly when there is recurrent infection or bleeding or a suspicion of malignancy. Despite satisfactory resolution of symptoms and eradication of the pseudocyst, splenectomy is associated with potentially long-lived complications, including an increased risk of post-splenectomy infection, thromboembolic events, and immune alterations, justifying immunization and the use of prophylactic antibiotics. The management of splenic pseudocysts also represents a problem that requires careful consideration of the patient as a whole, cyst characteristics, and available treatment modalities, while taking into account a multidisciplinary team and informed decision-making for individualized care. Diagnosis and therapy of acute abdominal conditions can be performed with minimally invasive techniques, i.e., laparoscopy [4].

The successful management of splenic pseudocysts necessitates a holistic approach, integrating clinical acumen, advanced imaging techniques, and tailored therapeutic interventions to optimize patient outcomes and mitigate potential complications. Given the rarity of splenic pseudocysts and their heterogeneity of etiologies, along with the absence of a typical clinical presentation, splenic pseudocysts pose a diagnostic and therapeutic dilemma for clinicians. It is, therefore, important that healthcare professionals are aware of the significance of maintaining a broad understanding of splenic pseudocysts, as this requires a high degree of suspicion in clinical settings involving splenic pseudocysts [5], the ability to undertake advanced imaging interpretation adequately, and the ability to tailor management strategies according to individual patient needs to improve diagnostic accuracy, therapeutic efficacy, and patient-centered care. Further, acute and chronic pseudocysts must be differentiated, as they have different natural histories and treatment approaches. Ultrasound is the initial modality used to detect abdominal masses. The well-defined, hypoechoic lesion may appear as a pseudocyst [4]. CT scans provide more detailed imaging, giving a clear image of the size and location of the pseudocyst, along with its characteristics and the relationship of the pseudocyst with other abdominal structures. MRI can be used in complicated cases where there is clearer delineation of the contents of the cyst and surrounding structures. The use of endoscopic ultrasound may be helpful in some cases, especially when other diagnostic methods are inconclusive. Management of splenic pseudocysts depends on their size, symptoms, and risk of complications [5]. Therefore, small asymptomatic pseudocysts may be observed without further intervention. However, regular imaging follow-ups are suggested to monitor for changes in size and/or the appearance of complications.

Percutaneous drainage may be an option in cases of large, symptomatic pseudocysts or those at risk for rupture. This involves needle drainage of the cyst under imaging guidance. In cases of large, symptomatic pseudocysts, splenectomy or partial splenectomy may be required if there is a risk of rupture. In rare cases, endoscopic approaches may be considered to drain or decompress the pseudocyst.

Treatment for splenic damage is usually conservative, if possible, to prevent complications post-splenectomy [6]. Subcapsular or pericapsular hematomas occur frequently among patients and may either liquefy and rupture later or resolve entirely. According to Black et al., a subcapsular hematoma in a hemodynamically stable patient is neither an indication of delayed splenic rupture nor a sign that surgery is required [9]. Around 90% of delayed ruptures occur within the first four weeks, and approximately 70% happen within the initial two weeks. A resolved subcapsular or intraparenchymal hematoma may occasionally become encased in fibrous tissue, which eventually liquefies to form a pseudocyst. About three-quarters of all splenic non-parasitic cysts are pseudocysts. They may hold up to 3 L of turbid, dark fluid while becoming quite large. The lining of pseudocysts is not epithelial. They may have thick or thin fibrous walls, and the fibrous capsule frequently shows signs of chronic inflammation.

The primary complaint is upper-left quadrant pain that extends to the left shoulder. Loss of appetite and a sense of fullness after meals may result from pressure on the stomach. Physical examination frequently reveals a smooth mass in the upper-left quadrant. Over time, some pseudocysts enlarge and may rupture spontaneously or in response to additional trauma. Conservative treatment and follow-up scans are recommended for individuals with splenic hematomas until the hematoma completely resolves. This allows for the identification of patients who may subsequently develop pseudocysts. A calcified mass in the left upper quadrant of the abdomen may be visible on a standard X-ray. While a CT scan can diagnose splenic rupture, it cannot be used to predict delayed rupture. The primary diagnostic method in acute cases is peritoneal lavage, which has a 95%-99% accuracy rate for diagnosing hemoperitoneum. CT scans provide crucial information regarding the volume of hemoperitoneum, the degree of splenic rupture and hematoma, the extent of parenchymal injury, hilar involvement, and any associated intraperitoneal or retroperitoneal injuries. This data can be extremely beneficial when determining whether conservative treatment is appropriate. According to Fabian et al., CT scans can diagnose blunt abdominal injuries with 85% sensitivity, 100% specificity, and 97% accuracy [10]. Ultrasonography is also helpful in diagnosing organ damage and detecting blood in the abdominal cavity. It is particularly valuable when monitoring patients with multiple injuries requiring orthopedic traction or immobility. The natural history of post-traumatic splenic pseudocysts is largely unknown in the absence of symptoms, and they remain rare. However, unless the cyst is small and resolving spontaneously, most patients are diagnosed based on symptoms and require surgical intervention. Large, central, multifocal cysts and complications - including rupture, abscess formation, or bleeding - require splenectomy. In many cases, it is sufficient to remove the cyst and secure the edges of the excision. This technique is often performed laparoscopically to identify the weakest portion of the cyst wall [11], with or without the assistance of an ultrasound probe.

## Conclusions

Splenic pseudocysts are benign lesions, typically resulting from splenic injury - either due to trauma or often overlooked injuries - and subsequent hematoma resorption. Preoperative differentiation from other cystic lesions can be challenging. While most cases are asymptomatic, symptomatic pseudocysts may require surgical intervention. Whenever possible, spleen-preserving techniques are favored over radical surgical procedures, except in cases where the cyst is very large, involves the entire spleen, or is located at the splenic hilum. Patients should be vaccinated against pneumococcal, meningococcal, and *Haemophilus influenzae* type b (Hib) in accordance with the Centers for Disease Control and Prevention (CDC) schedule for post-splenectomy vaccination, either preoperatively or postoperatively.
